# Supplementary material for: Particulate matter emission sources and meteorological parameters combine to shape the airborne bacteria communities in the Ligurian coast, Italy
Source: Sci Rep. 2021 Jan 8;11:175. doi: 10.1038/s41598-020-80642-1 (PMC7794459; doi:10.1038/s41598-020-80642-1)
Supplement: Supplementary file 1 — Supplementary Information 1. [file 41598_2020_80642_MOESM1_ESM.pdf]

Particulate Matter emission sources and meteorological parameters combine to shape the airborne bacteria communities in the Ligurian coast, Italy

Giorgia Palladino, Pietro Morozzi, Elena Biagi, Erika Brattich, Silvia Turroni, Simone Rampelli, Laura Tositti, Marco Candela

marco.candela@unibo.it

SUPPLEMENTARY MATERIALS

Supplementary Table S1 (2 pages) –Normalized contributions per sample of the seven factors resolved by PMF analysis. The first column reports the sample ID. All the other columns represent the contribution of each factor identified by PMF on the corresponding sample.

Sample ID	Factor 1	Factor 2	Factor 3	Factor 4	Factor 5	Factor 6	Factor 7
1	-0.01	-0.12	2.37	1.7	-0.2	2.96	-0.2
2	0.03	-0.2	3.73	1.17	0.18	3.04	-0.14
3	2.83	0.47	3.42	1.22	0.13	4.42	-0.2
4	4.17	1.34	2.84	1.24	0.31	5	0.18
5	0.89	-0.2	4.4	3.68	-0.2	3.34	0
6	0.94	0.96	0.98	1.96	-0.16	1.2	0.24
7	0.16	1.74	0.9	0.9	0.29	0.54	0.71
8	1.25	0.97	3.39	-0.2	1.21	0.55	0.59
9	-0.2	0.93	2.79	0.18	1.6	0.8	0.88
10	0.67	-0.2	0.28	-0.09	1.01	9.02	-0.07
11	2.29	0.09	-0.02	-0.2	0.79	5.42	-0.08
12	0.31	0.36	2.42	0.48	2.25	1.42	0.1
13	0.61	1.23	0.41	0.93	0.98	1.61	-0.01
14	1.9	2.14	0.3	0.11	0.4	2.38	-0.14
15	0.02	2.57	2.02	0.68	0.67	0	0.2
16	-0.05	0.36	1	0.65	-0.04	1.29	-0.09
17	1.74	0.28	3.35	0.39	0.14	0.9	-0.09
18	1.89	0.17	2.61	0.52	0.44	1.38	-0.13
19	0.75	0.38	3.12	0.34	0.57	1.39	-0.08
20	1.03	1.98	3.08	0.17	0.97	1.21	0.03
21	0.08	1.7	8.18	0.63	0.18	0.4	0.06
22	0.08	1.38	0.37	0.17	0.85	0.33	3.16
23	2.76	1.06	0.18	0.58	0.49	1.94	-0.2
24	1.58	3.06	1.85	1.71	0.54	2.5	-0.1
25	2.27	2.17	0.76	2.56	0.29	0.49	0.04
26	3.26	0.93	1.15	1.32	0.58	0.96	0.15
27	2.36	1.71	-0.2	1.7	-0.05	1.98	-0.17
28	1.71	3.84	-0.16	1.53	-0.2	3.08	-0.14
29	1.24	1.52	1.83	2.36	0.34	1.53	-0.03
30	4.01	0.15	1.05	-0.2	2.21	3.42	0.64
31	2.65	0.27	1.74	0.04	2.18	1.61	0.31
32	0.68	-0.1	4.53	1.13	0.46	1.42	0
33	0.12	0.99	0.41	0.51	0.26	0.11	0.13
34	1.32	0.35	0.08	0.39	0.12	0.3	0.43
35	0.54	0.72	0.53	0.34	0.41	0.29	0.65
36	-0.18	1.84	1.21	0.72	0.38	0.9	0.07
37	0.18	0.84	-0.16	0.09	-0.04	0.94	-0.05
38	0.17	0.79	0.7	-0.18	0.88	0.42	2.75
39	0.49	0.42	0.39	0.32	0.63	0.74	0.24
40	-0.13	0.57	0.01	0.4	-0.04	0.33	-0.01
41	-0.19	0.59	0.21	0.37	0.17	0.25	0.22
42	0.54	0.68	0.04	0.46	-0.05	0.3	-0.01
43	0.24	1.08	0.28	0.51	-0.1	0.75	0.33
44	0.18	1.94	0.83	0.01	0.81	1.26	3.35
45	0.14	0.66	0.09	0	-0.07	0.37	8.38
46	0.21	0.38	0.27	-0.12	0.79	0.13	6.81
47	0.26	0.6	0.61	-0.06	1.3	0.94	3.99

Sample ID	Factor 1	Factor 2	Factor 3	Factor 4	Factor 5	Factor 6	Factor 7
48	0.57	0.52	0.78	0.32	1.47	1.01	0.35
49	0.09	1.91	0.2	1.1	2.04	1.1	0.03
50	0.74	1.13	0.77	0.73	0.81	-0.2	0.82
51	3.12	1.29	-0.2	0.33	0.13	0.38	0
52	1.45	0.79	-0.1	0.4	0.26	0.73	0.08
53	1.62	2.78	0.21	0.34	0.31	0.59	-0.03
54	2.19	1.91	1.53	0.76	0.02	0.14	-0.02
55	0.74	0.5	4.04	0.58	0.11	-0.2	0.07
56	0.29	1.16	0.13	0.13	0.17	0.74	0.78
57	0.02	1.74	1.17	0.05	1.48	0.29	0.41
58	0.95	1.6	0.19	1.76	0.04	1.43	-0.09
59	0.25	1.47	0.18	1.9	-0.14	1.32	-0.08
60	-0.2	1.22	-0.06	2.94	0.44	0.7	-0.03
61	0.31	0.31	-0.2	1.71	0.62	1.08	-0.04
62	-0.16	-0.2	-0.2	3.74	2.36	0.79	0.13
63	0.13	0.85	0.23	0.94	1.11	0.07	2.08
64	0.11	0.83	1.31	0.08	1.99	0.05	1.68
65	0.34	0.53	0.57	-0.19	2.85	0.05	0.73
66	0.07	0.22	1.65	0.53	2.55	0.28	4.15
67	0.29	0.5	0.37	0.47	2.27	0.36	6.23
68	0.39	0.48	-0.2	0.2	0.62	0.27	8.04
69	0.17	0.31	0.53	-0.2	2.2	0.15	7.55
70	0.05	-0.2	1.14	0.18	1.92	0.18	5.54
71	0.31	1.37	0.66	0.44	2.36	0.48	0.11
72	1.23	1.38	0.3	1.66	1.22	0.65	-0.01
73	0.45	2.44	0.27	1.88	0.72	0.18	0.04
74	1.87	2.97	0.22	2.89	0.06	0.24	0
75	2.48	2.24	-0.11	2.57	0.1	1.39	-0.04
76	3.19	0.44	0.84	1.61	3.58	0.29	0.28
77	3.34	0.38	-0.1	3.68	2.98	0.62	-0.06
78	1.3	0.68	0.44	3.58	3.21	0.75	0.05
79	1.12	1.1	-0.02	3.3	2.47	0.92	0.01
80	0.13	0.74	0.88	2.93	2.8	-0.01	0.06
81	0.85	2.22	0.23	2.4	2.13	0.11	0.02
82	2.44	1.31	-0.16	0.23	1.18	1.02	-0.13
83	0.73	1.96	0.37	2.24	0.72	0.37	-0.02
84	3.45	0.53	-0.18	2.88	1.04	0.31	-0.11
85	1.6	0	1.45	0.92	1.27	0.53	3.41
86	2.4	1.89	0.08	1.78	0.36	0.22	-0.06
87	0.77	0.22	0.75	-0.16	3.71	0.55	0.34
88	0.48	0.76	0.01	1.72	1.81	0.2	0.01
89	0.19	-0.01	1.21	1.13	2.83	-0.2	0.38
90	0.33	0.06	1.22	1.24	3.15	0.26	0.45
91	0.28	0.53	1.51	-0.17	1.29	-0.09	9.64
92	0.24	-0.01	1.75	1.49	0.83	-0.02	5.21
93	0.43	0.45	0.74	-0.05	2.12	0.07	6.8
94	3.51	1.76	-0.03	0.42	0.44	0.63	0.36
95	2.07	2.15	0.02	0.62	0.51	0.6	0
96	1.37	2.47	0.11	1.42	0.36	0.48	-0.05
97	0.03	0.76	0.98	1.54	2.1	-0.2	0.19
98	0.2	0.12	0.45	1.93	2.33	0.76	0.07

Supplementary Table S2 (2 pages) – Meteorological parameters during the PM sampling period. The first column reports the sample ID, while the second indicates the sampling date. The meteorological parameters taken into account are temperature (T, °C), relative humidity (RH, %), pressure (P, mbar), rainfall (Rain, mm), wind speed (ws, m/s, and wind direction (wd, °). All values were taken every 30 min and averaged on a daily basis.

Sample ID	Sampling date	T (°C)	RH (%)	P (mbar)	Rain (mm)	ws (m/s)	wd (°)
1	01-Feb-2012	0.2	62.5	1019.3	0	4.6	318
2	05-Feb-2012	-1.6	49.1	1029.3	0	2.3	316
3	06-Feb-2012	-1.1	41.1	1023.5	0	3.7	321
4	09-Feb-2012	5.6	30.5	1025.5	0	1.9	323
5	11-Feb-2012	-0.8	47.9	1024	0	3	323
6	14-Feb-2012	4.5	41.2	1020.3	0	0.9	315
7	15-Feb-2012	5.2	62.7	1016.6	0	0.5	242
8	17-Feb-2012	8.5	61.9	1027.2	0	0.5	219
9	18-Feb-2012	11.3	67.8	1026	0	0.8	237
10	19-Feb-2012	8.8	73.4	1023.7	0	0.1	270
11	23-Feb-2012	13.6	32.4	1028.4	0	1.9	318
12	25-Feb-2012	12.1	73.2	1026.6	0	0.2	197
13	26-Feb-2012	13	52.1	1019.3	0	0.6	35
14	27-Feb-2012	11.5	40.2	1026.3	0	0.7	318
15	28-Feb-2012	10.7	72.4	1028	0	0.4	232
16	06-Mar-2012	6.9	64.7	1023.5	1	3.7	314
17	09-Mar-2012	11.5	42.1	1035.4	0	4.2	306
18	10-Mar-2012	12.4	30.8	1034.6	0	3	324
19	11-Mar-2012	14.8	26.1	1026.9	0	2.5	317
20	12-Mar-2012	13.8	60	1025.3	0	0.5	334
21	14-Mar-2012	13.3	64.4	1031.5	0	0.8	326
22	19-Mar-2012	12.3	73	1028.7	1.4	0.7	150
23	21-Mar-2012	17	37.3	1037.4	0	3.4	313
24	23-Mar-2012	13.7	58.8	1031.8	0	0.4	92
25	24-Mar-2012	14.9	50.7	1028.5	0	0.5	358
26	25-Mar-2012	16	45	1029.2	0.8	0.7	350
27	27-Mar-2012	17.4	36.8	1031.3	0	0.7	260
28	28-Mar-2012	16.3	44.6	1029.7	0	0.3	79
29	29-Mar-2012	13.9	65.2	1024	0	0.4	76
30	02-Apr-2012	14.6	53.2	1017	0	1.9	330
31	03-Apr-2012	15	65.6	1015.2	0	0.5	67
32	04-Apr-2012	13.6	78.2	1014	23.4	0.3	307
33	05-Apr-2012	14.6	71.2	1013.7	4.6	0.8	279
34	08-Apr-2012	15.4	44.4	1006.9	0	0.9	42
35	09-Apr-2012	11.3	49.4	1017.1	0	0.3	303
36	10-Apr-2012	10.5	79.4	1015.9	5.2	0.1	61
37	11-Apr-2012	10	68.9	1006.6	18.6	1.6	313
38	12-Apr-2012	11.6	75.4	1009.5	0	0.3	115
39	13-Apr-2012	14.8	59.9	1005.5	8.8	1	304
40	14-Apr-2012	12.5	68.3	1001.5	0.6	1.5	306
41	15-Apr-2012	12.7	70.2	1003.3	0.8	0.4	38
42	16-Apr-2012	14	57.6	1009.7	1.2	1.2	305
43	17-Apr-2012	15.5	50.2	1011.7	0.2	0.6	272
44	18-Apr-2012	13.6	70.3	1004.1	0.6	0.5	95
45	23-Apr-2012	NA	NA	1012.7	0	0.1	270
46	25-Apr-2012	11.5	80.7	1016.4	0	0.3	252

Sample ID	Sampling date	T (°C)	RH (%)	P (mbar)	Rain (mm)	ws (m/s)	wd (°)
47	26-Apr-2012	NA	NA	1024.2	0	NA	NA
48	02-May-2012	14.2	79.7	1022.8	0.2	0.3	68
49	10-May-2012	18.1	71.9	1029.4	0	0	68
50	14-May-2012	16.3	51.3	1020.1	0	1.2	287
51	16-May-2012	18.2	29.6	1017.4	0	0.7	56
52	17-May-2012	16.9	27.3	1022.9	0	0.5	268
53	18-May-2012	16.2	51.4	1021.4	0	0.1	354
54	19-May-2012	15.8	54.2	1020.8	0.4	0.1	277
55	20-May-2012	14.8	71.3	1017.5	11.4	0.3	291
56	22-May-2012	17.4	59.6	1012.3	0	0.2	256
57	23-May-2012	18.2	72.9	1019	0	0.1	68
58	26-May-2012	20.8	56.6	1021.4	0	0.2	276
59	27-May-2012	20.5	64	1022.1	0	0.1	149
60	30-May-2012	20.4	75.5	1022.9	0	0.3	236
61	02-Jun-2012	19.7	85.8	1021.2	1.2	0.3	79
62	03-Jun-2012	20	86.8	1019.9	5.4	0.5	73
63	04-Jun-2012	20.4	77	1013.4	1	0.4	181
64	05-Jun-2012	19.5	71.1	1016.9	0	0.3	68
65	06-Jun-2012	18.6	78.6	1018.6	0	0.2	68
66	09-Jun-2012	21.3	75.3	1017.3	0	0.1	231
67	11-Jun-2012	20.8	74.7	1009.6	0	0.2	69
68	12-Jun-2012	19.6	72.8	1007.7	0	0.6	64
69	13-Jun-2012	19.7	71.1	1016.6	0	0.6	67
70	14-Jun-2012	20	69.9	1023.3	0	0.7	69
71	15-Jun-2012	20	62.7	1026.1	0	0.2	68
72	16-Jun-2012	25.3	51.7	1025.1	0	0.2	36
73	17-Jun-2012	24.2	55.3	1023.5	0	0.1	68
74	19-Jun-2012	25.8	61.8	1020.7	0	0.1	63
75	20-Jun-2012	24.6	58.3	1018.5	0	0.1	293
76	21-Jun-2012	23.3	66.9	1015.9	0	0.4	63
77	22-Jun-2012	24.1	71.4	1019.9	0	0.3	66
78	23-Jun-2012	24.9	69.3	1022.9	0	0.2	85
79	24-Jun-2012	24.8	70.2	1022.4	0	0.2	80
80	25-Jun-2012	23.6	74.9	1018	0	0.2	68
81	26-Jun-2012	25.2	66.2	1019.8	0	0.1	62
82	27-Jun-2012	28.6	49.1	1021.3	0	0.3	14
83	28-Jun-2012	26.6	58.5	1019.3	0	0.2	68
84	01-Jul-2012	25.8	71.2	1019.2	0	0.2	59
85	03-Jul-2012	23.4	68.8	1020.3	0	0.3	68
86	05-Jul-2012	24.5	61	1016.3	0	0.1	60
87	07-Jul-2012	23.9	76.4	1018.4	0.6	0.6	66
88	08-Jul-2012	24.4	73.5	1017.8	0	0.3	69
89	10-Jul-2012	25.4	71.9	1017.6	0	0.4	72
90	11-Jul-2012	24.9	74.3	1018.4	0.2	0.4	68
91	13-Jul-2012	23.6	69.7	1015	0	0.3	68
92	14-Jul-2012	23.7	76.5	1013.6	0	0.8	71
93	15-Jul-2012	23.5	68.4	1015.7	0	0.3	68
94	16-Jul-2012	25.7	39.8	1024	NA	0.8	281
95	17-Jul-2012	25.4	44	1025.9	NA	0.3	78
96	18-Jul-2012	24.8	55	1023.6	NA	0.3	69
97	19-Jul-2012	21.3	76.7	1021	NA	0	68
98	20-Jul-2012	0.2	62.5	1019.3	0	4.6	318

Supplementary Table S3 (provided as Excel file) – Characteristics of the OTUs accounting for the compositional specificity of the four AM clusters. For each OTU, the following information is given: unique OTUs ID, taxonomy as assigned with SILVA database, the cluster/s to which each OTU is significantly correlated (i.e. the cluster/s in which the given OTU is significantly more represented), the BLAST best hit resulting from blasting OTU fasta sequences against the NCBI 16S rRNA sequence database, the percentage of identity (ID (%)) and coverage (coverage (%)) between the OTU sequences and the corresponding best hit, and the isolation source of each best hit as reported in the GenBank database.

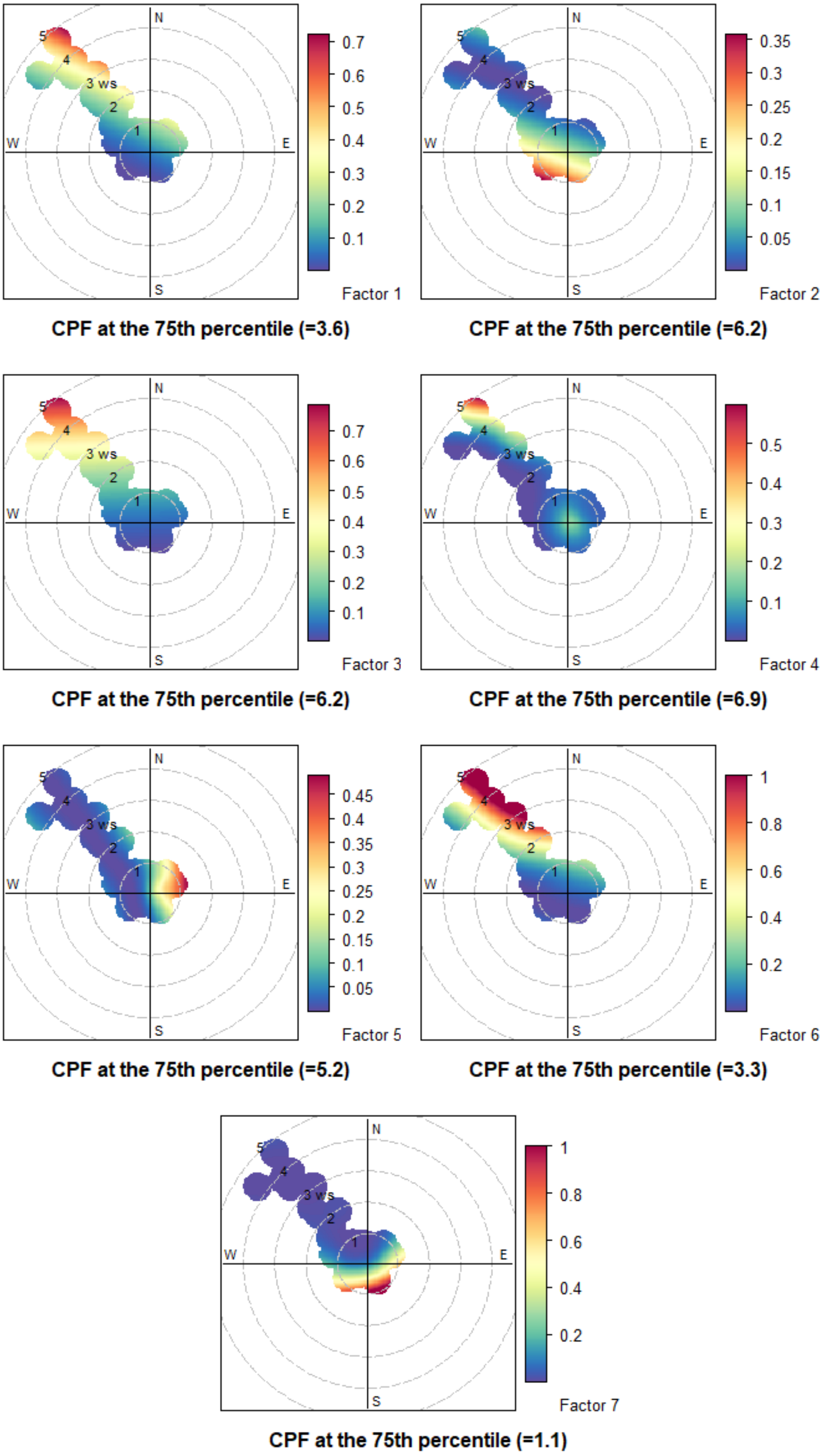

Supplementary Figure S1 – Association between the factors obtained by PMF analysis and the wind direction and intensity. Polar plots of the seven factors obtained by the PMF model. ws, wind speed; CPF, conditional probability function.

Supplementary Figure S2 - AM overall composition. Pie charts summarizing the microbiota composition of air filter samples at phylum (A) and family (B) level. Only phyla with relative abundance >1.5% in at least 10 samples and families with relative abundance >3% in at least 10 samples are shown.

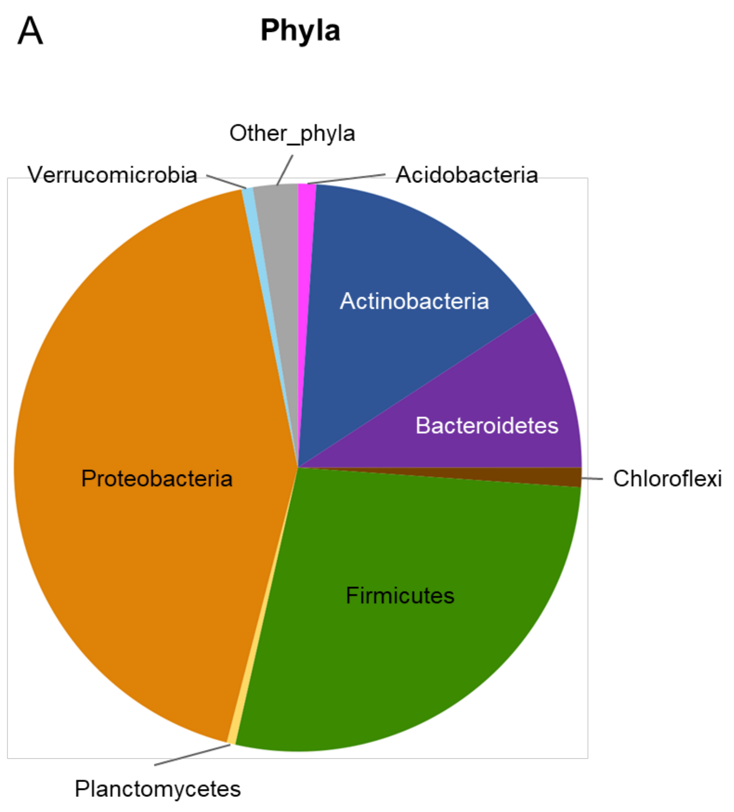

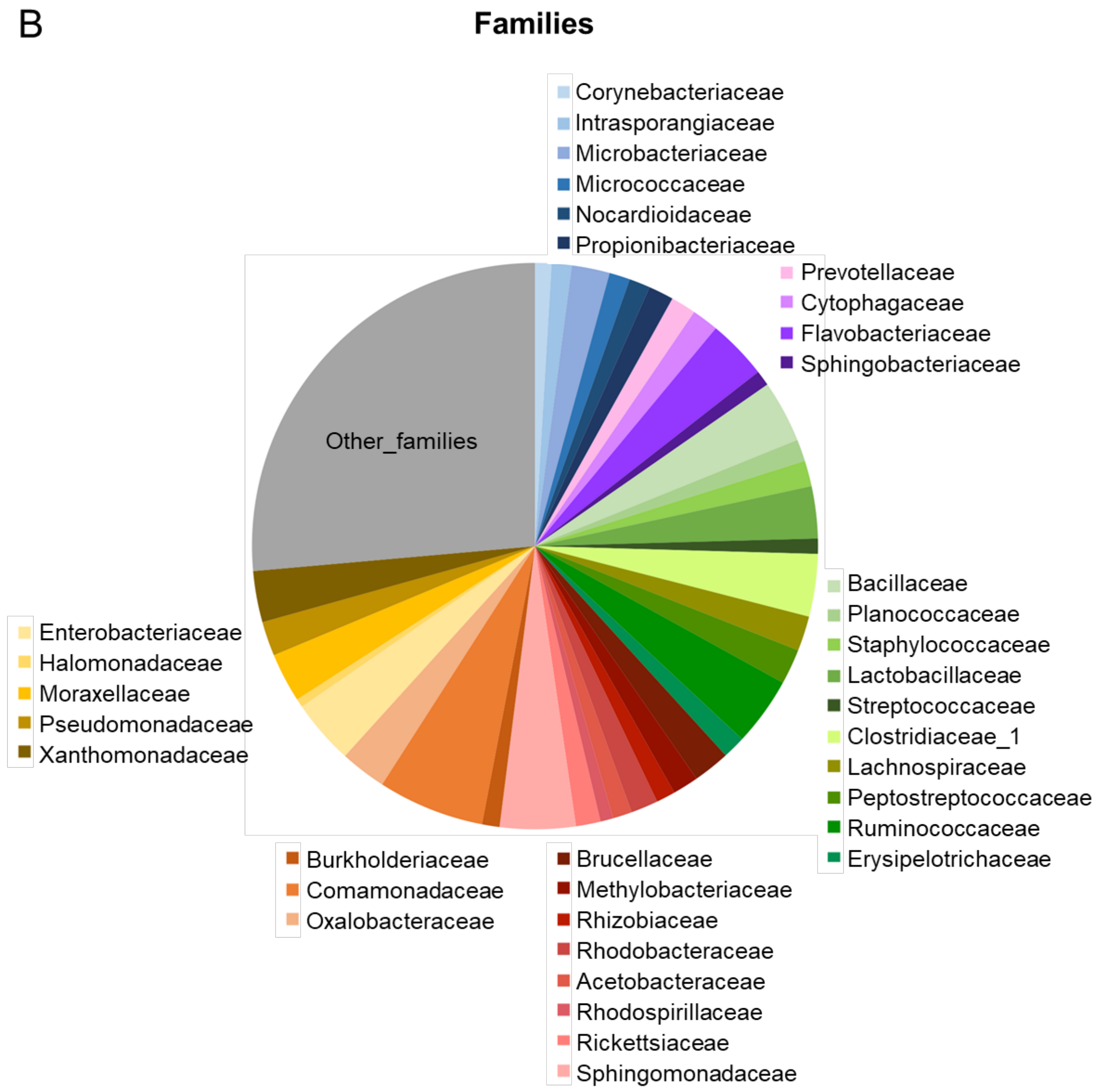

Supplementary Figure S3 – AM bacterial families differentially represented among the four microbial clusters. Box plots showing the bacterial families whose relative abundance is significantly differently distributed among the microbial clusters C1-C4 (Kruskal-Wallis test, FDR-corrected p-value ≤ 0.05*, p-value ≤ 0.01** and p-value ≤ 0.001***). The central box represents the distance between the 25th and 75th percentiles. The median is marked with a black line. Whiskers identify the 10th and 90th percentiles.

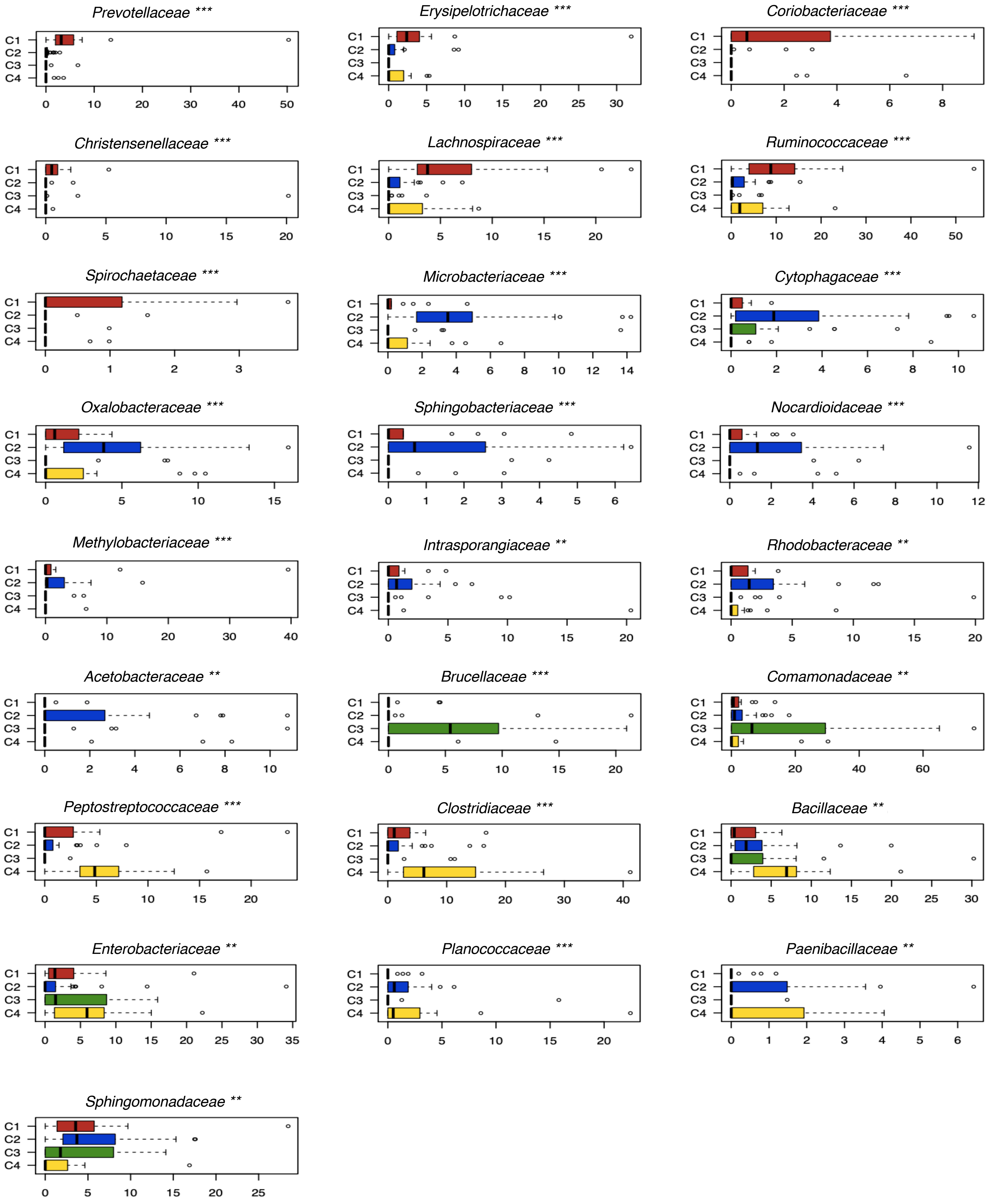

Supplementary Figure S4 (2 pages) – AM-associated OTUs showing different distribution across microbial clusters. Box plots showing the OTUs whose relative abundance is significantly differently distributed among the four microbial clusters C1-C4 (Kruskal-Wallis test, FDR-corrected p-value ≤ 0.05*, p-value ≤ 0.01** and p-value ≤ 0.001***). The central box represents the distance between the 25th and 75th percentiles. The median is marked with a black line. Whiskers identify the 10th and 90th percentiles. unc., unclassified; amb., ambiguous.

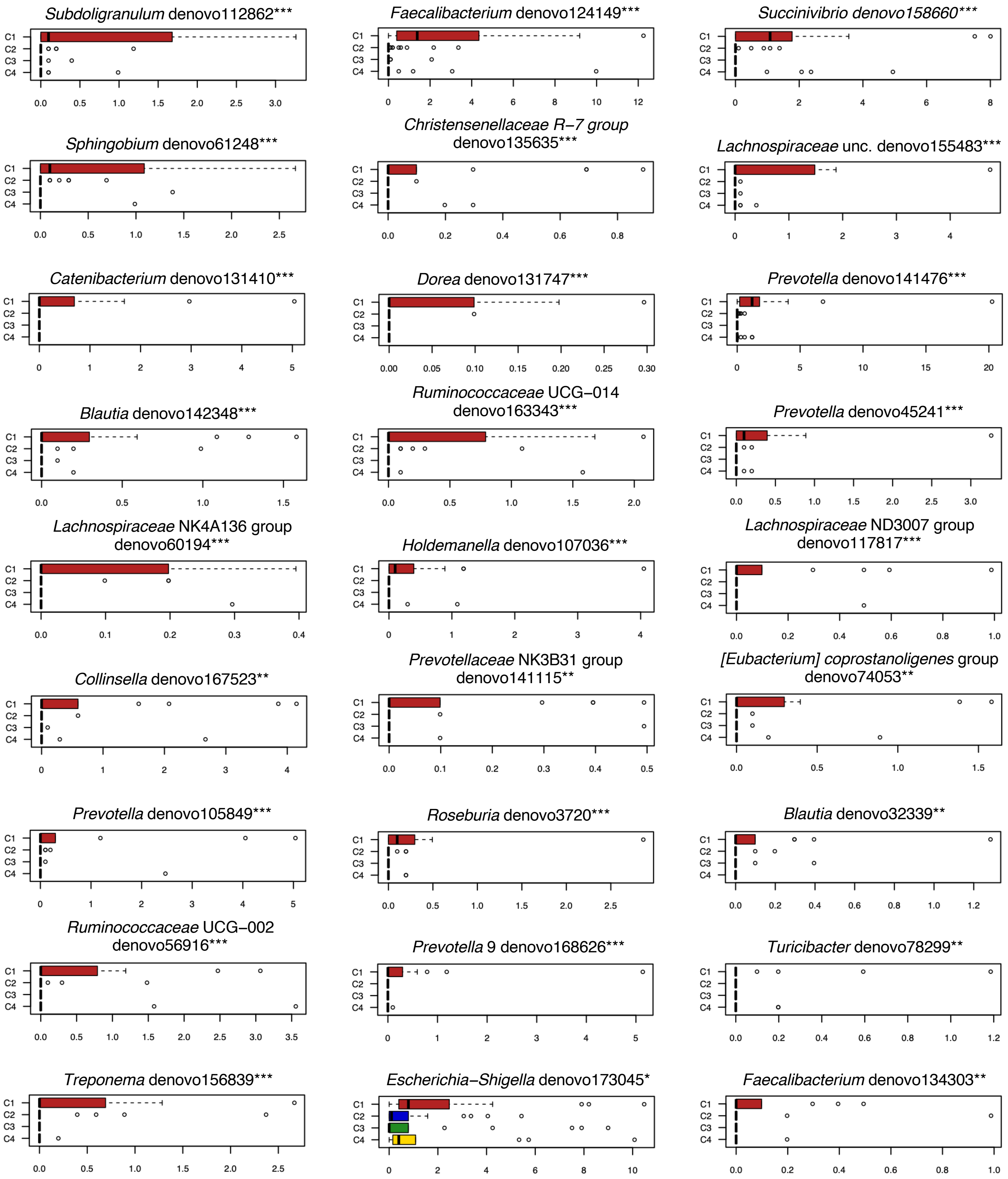

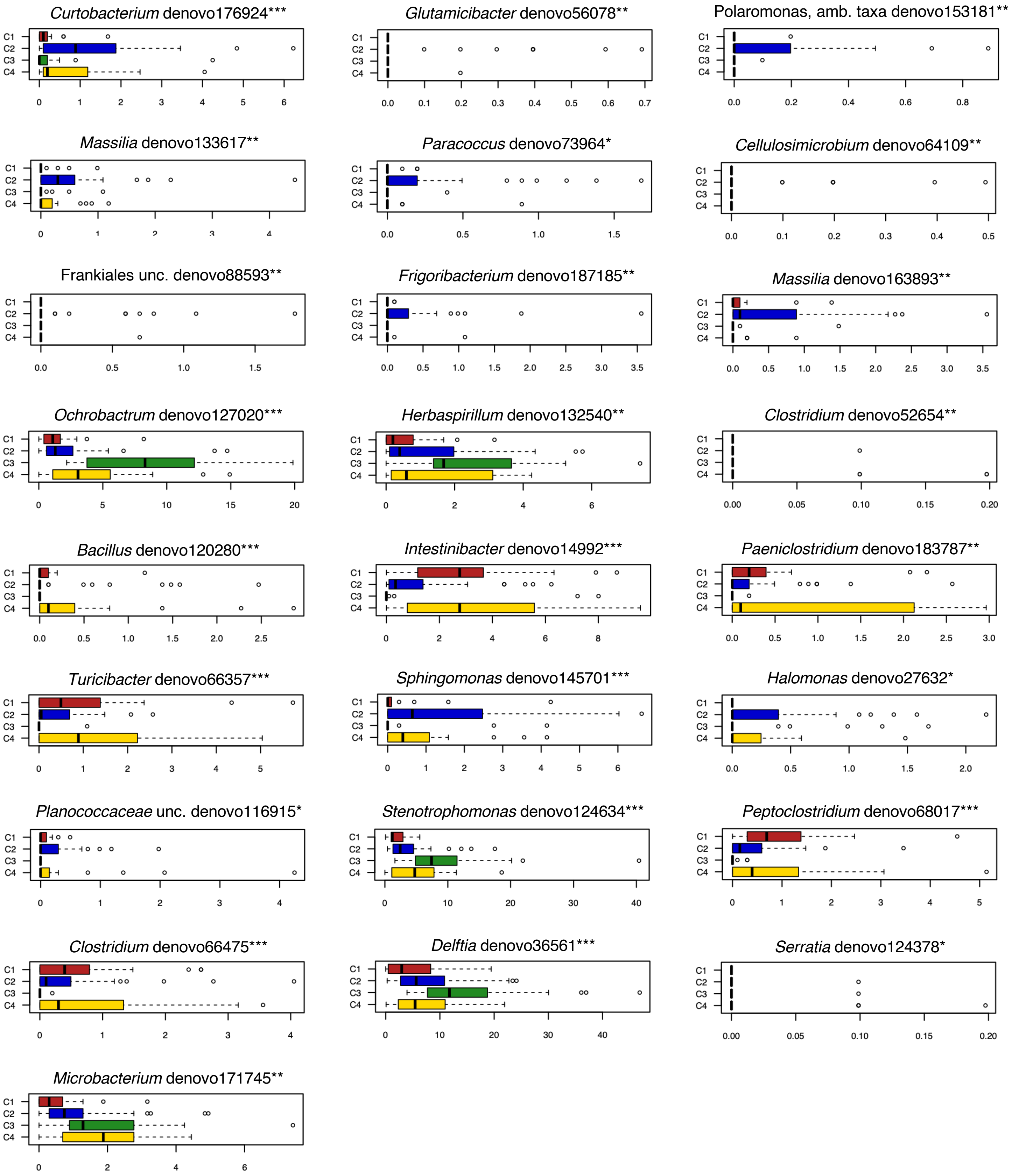

Curtobacterium denovo176924***
C1
C2
C3
C4
0 1 2 3 4 5 6
Glutamicibacter denovo56078**
0.0 0.1 0.2 0.3 0.4 0.5 0.6 0.7
Polaromonas, amb. taxa denovo153181**
0.0 0.2 0.4 0.6 0.8
Massilia denovo133617**
0 1 2 3 4
Paracoccus denovo73964*
0.0 0.5 1.0 1.5
Cellulosimicrobium denovo64109**
0.0 0.1 0.2 0.3 0.4 0.5
Frankiales unc. denovo88593**
0.0 0.5 1.0 1.5
Frigoribacterium denovo187185**
0.0 0.5 1.0 1.5 2.0 2.5 3.0 3.5
Massilia denovo163893**
0.0 0.5 1.0 1.5 2.0 2.5 3.0 3.5
Ochrobactrum denovo127020***
0 5 10 15 20
Herbaspirillum denovo132540**
0 2 4 6
Clostridium denovo52654**
0.00 0.05 0.10 0.15 0.20
Bacillus denovo120280***
0.0 0.5 1.0 1.5 2.0 2.5
Intestinibacter denovo14992***
0 2 4 6 8
Paeniclostridium denovo183787**
0.0 0.5 1.0 1.5 2.0 2.5 3.0
Turicibacter denovo66357***
0 1 2 3 4 5
Sphingomonas denovo145701***
0 1 2 3 4 5 6
Halomonas denovo27632*
0.0 0.5 1.0 1.5 2.0
Planococcaceae unc. denovo116915*
0 1 2 3 4
Stenotrophomonas denovo124634***
0 10 20 30 40
Peptoclostridium denovo68017***
0 1 2 3 4 5
Clostridium denovo66475***
0 1 2 3 4
Delftia denovo36561***
0 10 20 30 40
Serratia denovo124378*
0.00 0.05 0.10 0.15 0.20
Microbacterium denovo171745**
0 2 4 6
